# Optic nerve coloboma as extension of the phenotype of 22q11.23 duplication syndrome: a case report

**DOI:** 10.1186/s12886-020-01603-w

**Published:** 2020-08-17

**Authors:** Claudia Valencia-Peña, Paula Jiménez-Sanchez, Wilmar Saldarriaga, César Payán-Gómez

**Affiliations:** 1 Department of Ophthalmology, Faculty of Health, Universidad del Valle, Cali, Colombia; 2 Medical and surgery student, Universidad del Valle, Cali, Colombia; 3 Departments of Morphology and Gynaecology and Obstetrics, Universidad del Valle, Cali, Colombia; 4 Obstetrician Gynaecologist at Hospital Universitario del Valle, Cali, Colombia; 5 Department of Biology, Faculty of Natural Sciences, Universidad del Rosario, Bogotá, Colombia

**Keywords:** Coloboma of optic nerve, Duplication 22q11 q13, Bioinformatics, Array comparative genomic hybridization, Case report

## Abstract

**Background:**

22q11.2 duplication syndrome (Dup22q11.2) has reduced penetrance and variable expressivity. Those affected may have intellectual disabilities, dysmorphic facial features, and ocular alterations such as ptosis, hypertelorism, nystagmus, and chorioretinal coloboma. The prevalence of this syndrome is unknown, there are only approximately 100 cases reported. However Dup22q11.2 should have a similar prevalence of DiGeorge syndrome (1 in each 4000 new-borns), in which the same chromosomal region that is duplicated in Dup22q11.2 is deleted.

**Case presentation:**

We report a patient with intellectual disability, psychomotor development delay, hearing loss with disyllable pronunciation only, hyperactivity, self-harm, hetero-aggressive behaviour, facial dysmorphism, left facial paralysis, post-axial polydactyly, and for the first time in patients with Dup22q11.2, optic nerve coloboma and dysplasia in optic nerve. Array comparative genomic hybridization showed a 22q11.23 duplication of 1.306 million base pairs.

**Conclusions:**

New ocular findings in Dup22q11.2 syndrome, such as coloboma and dysplasia in the optic nerve, are reported here, contributing to the phenotypic characterization of a rarely diagnosed genetic syndrome. A complete characterization of the phenotype is necessary to increase the rate of clinical suspicion and then the genetic diagnostic. In addition, through bioinformatics analysis of the genes mapped to the 22q11.2 region, it is proposed that deregulation of the SPECC1L gene could be implicated in the development of ocular coloboma.

## Background

22q11.2 duplication syndrome (Dup22q11.2; Online Mendelian Inheritance in Man (OMIM) # 608363) is a genetic disorder with a dominant autosomal inheritance pattern, reduced penetrance and variable expressivity. The same chromosomal region involved in this syndrome is deleted in DiGeorge syndrome. Although both deletion and duplication are expected to occur in equal proportions as reciprocal events, very few duplications have been identified. While the calculated prevalence of DiGeorge syndrome is 1 of each 4000 new born, only around of 100 cases of Dup22q11.2 had been reported. This disparity in the prevalence of both syndromes could be explained at least in part because the phenotype of the duplication is not well understood.

In the majority of cases, the size of the duplication varies between 1.5 and 3.0 million base pairs (Mb). The phenotype is variable and has been reported to include intellectual disabilities, severe psychomotor development delays, language disorders, dysmorphic facial features and hypotonia; however, others with duplication do not present particular phenotypic findings [1].

In patients with Dup22q11.2, ocular findings such as ptosis, down-slanting palpebral fissures, epicanthal fold, hypertelorism, astigmatism, strabismus, hyperopia, myopia, nystagmus, chorioretinal coloboma, retinal vascular tortuosity and primary congenital glaucoma have been described [2].

Array comparative genomic hybridization (aCGH) is a molecular test that evaluates the entire genome and detects numerical and structural chromosome alterations throughout all chromosomes; this diagnostic technique surpasses karyotyping by light microscopy by finding deletions and duplications up to 10,000 times smaller. The aCGH is a test used in the differential diagnosis of patients with intellectual disabilities, psychomotor development delay, autism, and multiple congenital anomalies [3–5].

We report a patient with Dup22q11.2 with optic nerve coloboma and dysplasia in the contralateral optic nerve, findings not described in the reviewed literature, contributing to the phenotypic characterization of the syndrome and the epidemiology of this rare genetic disorder. In addition, through bioinformatics analysis of the genes mapped to the 22q11.2 region, deregulation of the SPECC1L gene could be implicated in the development of coloboma.

## Case presentation

A female patient 6 years and 3 months of age at the time of consultation, the product of a non-consanguineous relationship, was accompanied by her paternal grandmother, who had legal custody. According to the information provided by the caregiver, the patient was the product of a first pregnancy. At the time of conception, the father was 20 years old, and the mother, who was 18 years old, did not attend prenatal care appointments during pregnancy. She had a vaginal delivery without complications, but the new born required hospitalization for 3 days for phototherapy management of Rh incompatibility.

Regarding psychomotor development, the patient crawled at 16 months, walked at 3 years, and has spoken disyllables since 4 years of age. Currently, she does not have a comprehensible verbal language. In addition, she has hyperactivity disorder, has difficulty following instructions, displays self-harm and hetero-aggressive behaviour, for which she is treated pharmacologically with risperidone, and is enrolled in first grade, without meeting the learning requirements.

Among the relevant findings in the physical examination are weight, 16.3 kg (− 1.9 SD); height, 107 cm (− 1.9 SD); head circumference, 50 cm (− 1.64 SD); distance between outer canthi, 8 cm (50th percentile); distance between inner canthi, 2.6 cm (3rd to 25th percentile); interpupillary distance, 4.5 cm (25th percentile); wide *philtrum*, right palpebral ptosis, right ear with poor differentiation of the antihelix, left ear with prominent antihelix and hypoplastic lobe; short and wide neck; winging of the scapula occurs with myopathy of the muscles around the shoulder; high exit crease on palm; café-au-lait macule, approximately 6 cm in the hypogastrium; and hypotonia. A post-axial polydactyly surgical scar was found on the left hand. She had hypoacusis and only pronounced disyllables. During the consultation, the patient wandered spontaneously and showed signs of hyperactivity and a delay in psychomotor development, and intellectual disability became evident.

**Fig. 1:**
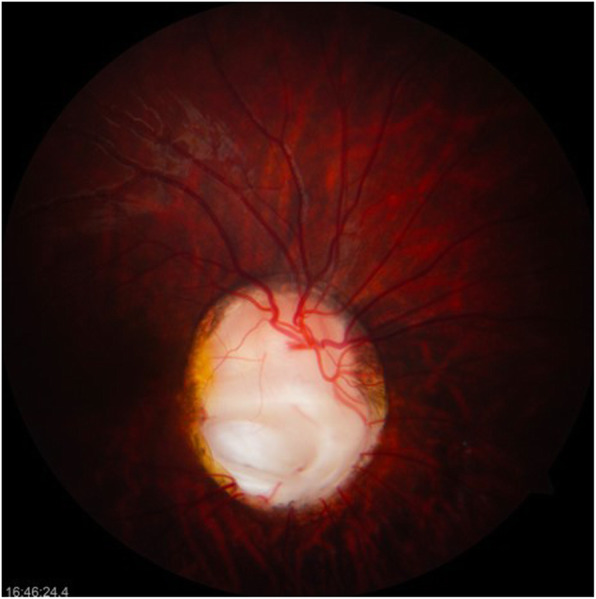
Photograph of the right optic disc. Optic nerve coloboma; the development of the inferior disc is worse than superior

**Fig. 2:**
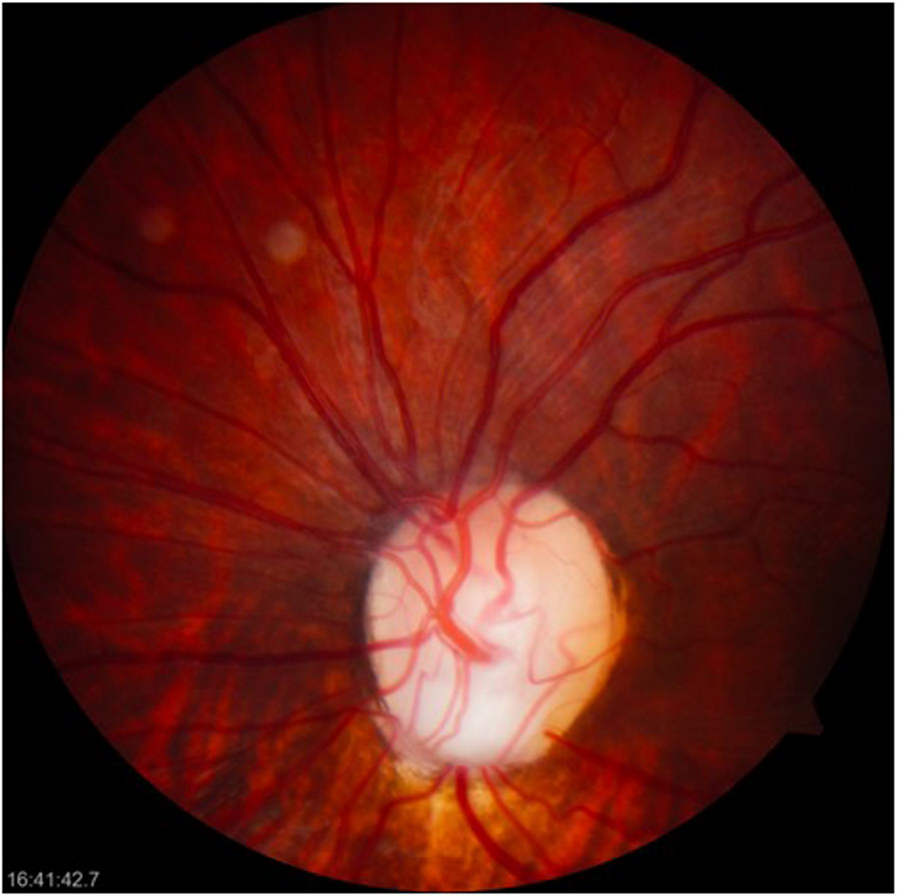
Photograph of the left optic disc. Forme fruste dysplastic optic disc with inferior coloboma

The following relevant findings were observed on ophthalmological physical examination: left facial paralysis, with secondary lagophthalmos of approximately 4 mm, with Bell’s phenomenon; and right palpebral ptosis with a palpebral fissure of 9 mm, unlike the left palpebral fissure of 12 mm. In the exploration of visual acuity, she fixed, followed and maintained with both eyes. The pupils had a diameter of 3 mm and were reactive to light, and no afferent pupillary defect was found. Extraocular movements were preserved, and the Hirschberg corneal reflex was centred. No significant findings were obtained in the exploration of the eye anterior segment, the cornea was clear, the anterior chamber was wide, the iris had a normal appearance, and crystalline lenses were transparent. Intraocular pressure measured by digital palpation was considered normal. Upon examination of the right fundus, a macrodisc was observed, with marked pallor and only a remnant of the superior part of the neuroretinal rim, marked peripapillary atrophy and the presence of an inferotemporal coloboma (Fig. 1). The left optic disc, of smaller size, had an excavation in the inferior position with absence of the inferior neuroretinal rim, and the remaining neural tissue was pink/orange and of normal aspect (Fig. 2). In both eyes, the macula had a normal appearance, and both retinas were attached.

The patient has been examined using multiple diagnostic aids, with alterations found in the following:

- At 5 months of age, electroretinography indicated a diffuse retinal anomaly in both eyes.
- In the eighth month, a patent foramen ovale with a left-to-right shunt was found on a two-dimensional echocardiogram, a finding managed by observation. The most recent echocardiogram at 6 years of age showed that the interauricular communication was closed, with persistence of the left superior vena cava.
- At 12 months of age, hearing potential tests identified profound hearing loss in the left ear and severe hearing loss in the right ear. The exam was repeated at 4 years of age, and moderate neurosensory hearing loss was found in both ears. Paediatric otolaryngology concluded that she was not a candidate for a cochlear implants, and hearing aids were utilized.
- At age 18 months, G-banding karyotyping indicated the chromosomal formula 46,XX. At that same age, a simple brain MRI scan showed – colpocephaly- a mild dilation of the posterior portion of the lateral ventricles.
- At 3 years of age, in a renal and urinary tract ultrasound, the left kidney (7.9 × 2.4 × 2.8 cm) was larger than the right kidney (5.7 × 2.2 × 2.3 cm), both without alterations in sonographic morphology. Four months later, videofluoroscopy showed a decrease in the oesophageal lumen at the T1 level.
- Electroencephalography showed moderate diffuse cortical dysfunction without epileptogenic characteristics. Using electromyography, severe axonal injury of the left facial nerve was observed.
- At 5 years, the patient was diagnosed with hypothyroidism, and treatment with levothyroxine was started, with an adequate therapeutic response.

Based on the history of the patient, the findings in the physical examination and the observed congenital anomalies, it was concluded that the origin was most likely genetic, and aCGH was requested. The result was a 22q11.23 duplication of 1.306 Mb (chr22: 23654163–24,959,827).

The grandmother, who was the legal guardian of the patient, signed an informed consent form allowing the child to be photographed and the use of medical history data.

## Discussion and conclusions

The first case of Dup22q11.2 syndrome was reported in 1999 by Edelmann et al., who described a patient younger than 4 years old with a duplication of 3.0 Mb. The main phenotypic characteristics described were delayed motor development, marked hypotonia, significant delay in language skills and mild facial dysmorphic features [6]. In 2003, Ensenauer et al. reported 13 new cases, most of them with duplications of 3.0 Mb, but broad phenotypic differences were found among the cases [7].

Dup22q11.2 syndrome is infrequent, and its prevalence has not been established. However, in recent years, with the increasing use of comparative genomic hybridization in patients with congenital anomalies, intellectual disabilities and autism, the diagnosis of this syndrome has increased; thus far, approximately 100 cases have been reported [8].

The chromosome region implicated in Dup22q11.2 syndrome is the same as that involved in DiGeorge syndrome or 22q11.2 deletion syndrome. The prevalence of the latter is 4:10,000 live births, and given that the genetic mechanism that causes the syndrome is also responsible for the duplication, it would be expected that this would occur in a similar proportion; however, that is not observed. This is because the deletion, whose penetrance is 100%, leads to the expression of a characteristic phenotype widely known that, although variable from mild to severe, leads to clinical suspicion and diagnostic testing, while in duplication, in addition to variable expression with a not yet clearly defined phenotype, incomplete penetrance is presented, which explains its under diagnosis [1, 9].

Dup22q11.2 syndrome is a product of chromosomal rearrangement that can also cause the deletion of that same locus because this region contains low copy repeats (LCRs) that are susceptible to non-allelic homologous recombination. Specifically, this locus has 8 LCRs, A through H, and depending on which of these have been deleted or duplicated, can be either a proximal -LCRs close to the centromere: A to D or distal -LCRs close to the telomere: E to H- deletion or duplication. The most common 22q11.2 duplications are those of 3.0 Mb, which involve the LCRs from A to D, and those of 1.5 Mb, which encompass LCRs from A to B [1, 10]. For the reported patient, her distal duplication is atypical because it implies duplication of 1.306 Mb with F-H LCRs.

In Dup22q11.2 syndrome, a variable phenotype has been described; even in members of a family with the same duplication, different phenotypes have been reported. The main non-ocular phenotypic characteristics of Dup22q11.2 syndrome include cognitive deficit, psychomotor development delay, decreased hearing, mild micro-retrognathia, cleft palate, elongated face, urogenital tract malformations, and congenital heart defects. Other less common phenotypic characteristics are hyperactivity, macro-microcephaly, seizures and skeletal defects [9, 11]. The patient reported here presented cognitive deficit, psychomotor development delay, hyperactivity, decreased hearing, patent foramen ovale, and persistent left superior vena cava. Additionally, she has alterations not previously described, such as left facial paralysis, dysplastic ears, and post-axial polydactyly. The characteristics found in the patient reported here show that the phenotype of Dup22q11.2 syndrome is not only variable but has not yet been described in its entirety.

In addition to the characteristics mentioned above, ocular involvement in Dup22q11.2 has been reported; the most frequently described findings are hypertelorism, down-slanting palpebral fissures, and sparse and thin eyebrows. Less frequently, palpebral ptosis, epicanthal fold, astigmatism, strabismus, hyperopia, myopia, nystagmus, chorioretinal coloboma, retinal vascular tortuosity, and primary congenital glaucoma have been described [2]. In the patient reported here, left-side enlarged palpebral fissure and lagophthalmos without keratopathy , most likely secondary to upper eyelid retraction, were found. Ophthalmology revealed a coloboma of the right optic nerve and dysplasia of the left optic nerve; these findings have not been described in the phenotype of Dup22q11.2 syndrome.

Cat eye syndrome (CES) is found in the differential diagnosis of Dup22q11.2 syndrome. Coloboma of the iris, retina and choroids, preauricular pits and appendages and anal atresia have been reported in those affected by CES; however, these characteristics are not consistently found in patients with CES. Common findings include intellectual disability, down-slanting palpebral fissures, cleft palate, congenital heart defects, renal and urinary tract abnormalities, and bone defects. The patient described here presented several of the phenotypic features of CES [12].

CES is caused in most cases by the presence of a bisatellite supernumerary marker chromosome, formed by two copies of the proximal part of chromosome 22, which includes satellites, the entire short arm, the centromere and part of the long arm corresponding to the 22q11 region. This additional chromosome, which occurs in mosaics in one-third of cases, usually has two centromeres and is the product of an inverted duplication 22q11, resulting in partial tetrasomy of this segment, whose distal limit is superimposed on the common distal deletion breakpoint of the 3 Mb deletion seen in 22q11.2 deletion syndrome [13, 14]. The patient reported here presented coloboma of the optic nerve, not of the retina, nor of the iris, and did not have other frequent characteristics of CES, such as anal atresia or preauricular abnormalities. In addition, the 22q11.23 duplication found in the patient was not included in the region involved in CES. Therefore, the patient did not meet the criteria for a CES diagnosis.

To understand the molecular pathways involved in optic nerve coloboma is necessary to review the embryologic development of the eye. The formation of the eye begins early in embryonic development, day 22, by interactions between the neuroectoderm, the superficial ectoderm and the mesenchyme, and the ocular fields are formed [15]. These appear in the neural plate and are determined by PAX-6, which in turn is regulated by SHH expression in the precordal plate [16]. On day 24 after the evagination of the prosencephalon, the optic vesicles appear. On day 28, the optic vesicles express bone morphogenetic protein 4 and 7 (BMP4 and BMP7) and induce thickening of the superficial ectoderm to form the lens placode. The placode expresses SIX-3, which activates the expression of SOX-2 and PAX-6 [16]. These last two proteins activate the expression of genes necessary for invagination of the placode and the formation of the crystalline lens. On day 32, the placode of the lens and the distal surface of the optic vesicle are invaginated, the lens vesicle is formed by the invagination of the first, and by the invagination of the second, the optic cup is formed, from which the retina and the epithelia of the ciliary body and iris will develop. The optic cup remains attached to the diencephalon by the optic stem. On the ventral surface of the optic cup and the optic stem, a groove develops that corresponds to the choroid fissure; through this fissure, the hyaloid artery and vein traverse to the lens and nourish the inner layer of the optic cup. PAX-2 is expressed in cells of the optic stem, while PAX-6 is expressed in the optic cup; these transcription factors are involved in the orientation of the axons of the glandular cells of the retina so that they are introduced in the optic stem and form the optic nerve. In week 5, the margins of the choroid fissure move the periocular mesenchyme and approach along the length of the stem and optic cup. The intercalation of positive SOX2/PAX2/Vimentin astrocytes in the developing fissure and optic nerve contribute to closure of the optic nerve [17]. By week 7, it is completely closed. If this process is not performed correctly, a coloboma is produced [18].

Although some environmental factors that could be associated with the development of colobomas have been described, it is believed that most have genetic causes [19]. At least 39 genes linked to the development of colobomas have been identified; in all of them, monogenic mutations have been reported as the cause of the disease [20]. Among these 39 genes, mutations in eight of them can produce optic nerve colobomas: PAX6, PAX2, RAX, GDF6, SEMA3E, IGBP1, BMP7 and PDE6D. None of these genes are mapped to the 22q11.23 region.

The noteworthy phenotypic characteristics of the patient with Dup22q11.23 are developmental delay, facial dimorphism and coloboma and optic nerve dysplasia. To determine which genes in the duplication could potentially explain these alterations, a bioinformatic analysis of the altered chromosomal region was performed.

**Fig. 3:**
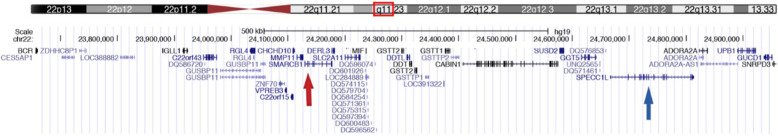
Chromosome 22, selected area q11.23, genomic coordinates 23.654.163–24.959.827. Genome browser from University of California Santa Cruz was used to interrogate the duplicated region in the patient. The red square on the long arm of ideogram of chromosome 22 show the compromised region. Bottom region of the figure shown the localization of known genes in the duplicated region as is annotated in GENCODE version 29, only one transcript is represented by each gene. SMARCB1 is pointed by red arrow and SPECC1L is pointed by blue arrow

In the 22q11.23 region, 66 genes are mapped between nucleotides 23.654.163–24.959.827, and 22 of these genes are annotated in the Gene Ontology gene function database [21] with diverse functions such as melanin synthesis, calcium signalling, glutathione metabolism and immune response, among others (Fig. 3). Five of the genes in the chromosomal region studied are registered and related to pathologies in the OMIM database [22] (Table 1).

**Table 1:** Genes that map to 22q11.23 and that are annotated in the OMIM database. Mutations in five genes in the 22q11.23 region have been reported as causal of Mendelian diseases

Gene	Disease in the OMIM database
CHCHD10	615,911 ~ Frontotemporal dementia and/or amyotrophic lateral sclerosis 2
CHCHD10	615,048 ~ Spinal muscular atrophy, Jokela type
CHCHD10	616,209 ~ Myopathy, isolated mitochondrial, autosomal dominant
MIF	604,302 ~ Rheumatoid arthritis, systemic juvenile, susceptibility to
SMARCB1	609,322 ~ Rhabdoid predisposition syndrome 1
SMARCB1	162,091 ~ Schwannomatosis-1, susceptibility to
SMARCB1	614,608 ~ COFFIN-SIRIS SYNDROME 3, Mental retardation, autosomal dominant 15
SMARCB1	609,322 ~ Rhabdoid tumours, somatic
SPECC1L	600,251 ~ Facial clefting, oblique, 1
SPECC1L	145,410 ~ Opitz GBBB syndrome, type II
UPB1	613,161 ~ Beta-ureidopropionase deficiency

According to disease phenotypes registered in the OMIM database, two genes with high potential of being related to the Dup22q11.23 phenotype were prioritized: SWI/SNF related, matrix associated, actin dependent regulator of chromatin, subfamily b, member 1 (SMARCB1) and sperm antigen with calponin homology and coiled-coil domains 1-like (SPECC1L).

The SMARCB1 protein is part of one of several subunits of the SWI/SNF complex that functions as a chromatin remodelling factor [23]. Mutations in this gene produce Coffin-Siris syndrome 3 (CSS3), which presents with developmental delay, coarse facial features, feeding difficulties and the absence of phalanges or hypoplastic fingernails of the fifth finger of the hand [24]. CSS3 has an autosomal dominant inheritance pattern, and because all mutations reported as causative of CSS3 are missense, it has been proposed that the disease is caused by a dominant negative effect or by gain-of-function effects [23]. The case reported, in addition to developmental delay, presented with post-axial polydactyly; it is tempting to speculate that the presence of three copies of the SMARCB1 gene will increase its transcription and therefore the availability of the protein in the cells to exert effects contrary to the heterozygous mutation of the gene.

Optic nerve coloboma, another of the prominent characteristics of the phenotype secondary to 22q11.23 duplication in the patient reported here, could be related to an increase in the gene dose of SPECC1L. This gene codes for the protein cytospin-A, which stabilizes microtubules. This protein is necessary for the migration of neural crest cells to form the forehead, the nasal bridge and the lower jaw. Mutations in the SPECC1L gene produce oblique facial clefting [25], and Opitz GBBB syndrome type II (GBBB2). Both diseases are autosomal dominant and are characterized by facial dimorphism with hypertelorism and facial clefts. Both syndromes, are described as including colobomas [25, 26].

For the development of the optic nerve and cup, concentration gradients of transcription factors (TFs), such as PAX2, PAX6, VAX1, VAX2, TBX2, TBX3, TBX5, VSX2 and MITF [20], are necessary. When looking for TF potentials in the promoter region of the gene SPECC1L by means of the TF2DNA tool [27], it was found that the gene promoter can be activated by PAX2 and PAX6. These two TFs are fundamental in the development of the eye [20], and mutations in them have been related to the presence of coloboma [28–31]. Due to the duplication of 22q11.23, the patient has three copies of the SPECC1L gene, and this increase in gene dosage could secondarily generate an increase in the expression level of this gene, altering the signalling pathways of PAX2 and PAX6 with the subsequent appearance of alterations in the optic nerve.

Although the exact causes of the patient findings cannot be determined by our analyses, they do allow us to propose that the increase in the number of copies of some genes from the 22q11.23 region could be directly related to the development of the abnormal phenotype. Specifically, we postulate alterations in SPECC1L with optic nerve coloboma as a potential transcriptional target of PAX2 and PAX6. Additional research is necessary to identify the exact mechanisms related to the abnormal phenotype caused by the duplication.

In conclusion, based in this case report, the ophthalmologic phenotype of 22q11.23 duplication syndrome should be expanded to additional ocular alterations as coloboma and dysplasia in the optic nerve. By the analysis of the genes in the duplicated region in patients with the syndrome, it is plausible that the triple genetic doses of SPECC1L gene could be involved in the development of ocular alterations by the deregulation of the PAX2 and PAX6 signalling pathways. Further research is necessary to confirm the previous hypothesis.

## Data Availability

All data supporting the discussion and conclusion of this article are presented in the manuscript.

## Abbreviations

aCGH: Array comparative genomic hybridization
BMP4: Bone morphogenetic protein 4
BMP7: Bone morphogenetic protein 7
CES: Cat eye syndrome
CSS3: Coffin-Siris syndrome 3
Dup22q11.2: 22q11.2 duplication syndrome
GBBB2: Opitz GBBB syndrome, type II
LCRs: Low copy repeats
Mb: Million base pairs
MRI Scan: Magnetic Resonance Imaging
OMIM: Online Mendelian Inheritance in Man
SD: Standard deviations
SMARCB1: SWI/SNF related, matrix associated, actin dependent regulator of chromatin, subfamily b, member 1
SPECC1L: Sperm antigen with calponin homology and coiled-coil domains 1-like
TFs: Transcription factors
